# Why #WeAreNotWaiting—Motivations and Self-Reported Outcomes Among Users of Open-source Automated Insulin Delivery Systems: Multinational Survey

**DOI:** 10.2196/25409

**Published:** 2021-06-07

**Authors:** Katarina Braune, Katarzyna Anna Gajewska, Axel Thieffry, Dana Michelle Lewis, Timothée Froment, Shane O'Donnell, Jane Speight, Christel Hendrieckx, Jasmine Schipp, Timothy Skinner, Henriette Langstrup, Adrian Tappe, Klemens Raile, Bryan Cleal

**Affiliations:** 1 Charité - Universitätsmedizin Berlin Department of Paediatric Endocrinology and Diabetes Berlin Germany; 2 Berlin Institute of Health Berlin Germany; 3 #dedoc° Diabetes Online Community Berlin Germany; 4 Population Health Sciences Royal College of Surgeons in Ireland Dublin Ireland; 5 Novo Nordisk Center for Biosustainability Technical University of Denmark Copenhagen Denmark; 6 #OpenAPS Seattle, WA United States; 7 School of Sociology University College Dublin Dublin Ireland; 8 The Australian Centre for Behavioural Research in Diabetes Melbourne Australia; 9 School of Psychology Faculty of Health Deakin University Geelong Australia; 10 Department of Psychology University of Copenhagen Copenhagen Denmark; 11 Department of Public Health, Section for Health Services Research University of Copenhagen Copenhagen Denmark; 12 AndroidAPS Hamilton New Zealand; 13 Diabetes Management Research Steno Diabetes Center Copenhagen Copenhagen Denmark

**Keywords:** diabetes, artificial pancreas, automated insulin delivery, open-source, patient-led, user-led, peer support, online communities, diabetes technology, digital health, mobile health, medical device regulation, motivation, sleep quality, do-it-yourself

## Abstract

**Background:**

Automated insulin delivery (AID) systems have been shown to be safe and effective in reducing hyperglycemia and hypoglycemia but are not universally available, accessible, or affordable. Therefore, user-driven open-source AID systems are becoming increasingly popular.

**Objective:**

This study aims to investigate the motivations for which people with diabetes (types 1, 2, and other) or their caregivers decide to build and use a personalized open-source AID.

**Methods:**

A cross-sectional web-based survey was conducted to assess personal motivations and associated self-reported clinical outcomes.

**Results:**

Of 897 participants from 35 countries, 80.5% (722) were adults with diabetes and 19.5% (175) were caregivers of children with diabetes. Primary motivations to commence open-source AID included improving glycemic outcomes (476/509 adults, 93.5%, and 95/100 caregivers, 95%), reducing acute (443/508 adults, 87.2%, and 96/100 caregivers, 96%) and long-term (421/505 adults, 83.3%, and 91/100 caregivers, 91%) complication risk, interacting less frequently with diabetes technology (413/509 adults, 81.1%; 86/100 caregivers, 86%), improving their or child’s sleep quality (364/508 adults, 71.6%, and 80/100 caregivers, 80%), increasing their or child’s life expectancy (381/507 adults, 75.1%, and 84/100 caregivers, 84%), lack of commercially available AID systems (359/507 adults, 70.8%, and 79/99 caregivers, 80%), and unachieved therapy goals with available therapy options (348/509 adults, 68.4%, and 69/100 caregivers, 69%). Improving their own sleep quality was an almost universal motivator for caregivers (94/100, 94%). Significant improvements, independent of age and gender, were observed in self-reported glycated hemoglobin (HbA_1c_), 7.14% (SD 1.13%; 54.5 mmol/mol, SD 12.4) to 6.24% (SD 0.64%; 44.7 mmol/mol, SD 7.0; *P*<.001), and time in range (62.96%, SD 16.18%, to 80.34%, SD 9.41%; *P*<.001).

**Conclusions:**

These results highlight the unmet needs of people with diabetes, provide new insights into the evolving phenomenon of open-source AID technology, and indicate improved clinical outcomes. This study may inform health care professionals and policy makers about the opportunities provided by open-source AID systems.

**International Registered Report Identifier (IRRID):**

RR2-10.2196/15368

## Introduction

### Background

Despite significant advances in health care, pharmaceuticals, and technological developments, type 1 diabetes remains a challenging chronic condition to manage, impacting life expectancy and diminishing quality of life [1-3]. Only a small proportion of people with type 1 diabetes achieve glycated hemoglobin (HbA_1c_) levels below 7.0% (58 mmol/mol), as recommended by therapeutic guidelines to reduce the risk of long-term diabetes-related complications [4-6]. The complexity of diabetes self-management bears a high cognitive load and can cause distress in everyday life, with approximately 40% of people with type 1 diabetes reporting distress and/or depressive symptoms, particularly prevalent among adolescents and young adults [7-10].

In addition to optimizing glucose levels and variability, diabetes technologies have the potential to ease complex decision making and thereby reduce the cognitive and emotional burden of diabetes self-management. The latest advances in diabetes therapy combine sensors for continuous glucose monitoring and insulin pumps with computerized control algorithms, thereby enabling automated adjustments to insulin delivery in response to the user’s changing glucose levels. Automated insulin delivery (AID) systems, also known as *artificial pancreas* or *(hybrid) closed-loop* systems, are in various iterations of development and automaticity. Although a variety of commercial AID systems are under development, and some have recently become available in a limited number of countries, they are not universally available, accessible, or affordable.

To fill in the gap, open-source AID systems, also called *Do-It-Yourself* Artificial Pancreas Systems (DIYAPS), have been created by people with diabetes, in the web-based community behind the hashtag #WeAreNotWaiting, with instructions and codes for these systems available freely and widely via open-source platforms. Although anyone can access this, each user has to take responsibility to build their individual system and use it at their own risk. Initial observational studies have described significant improvements in glycemic outcomes in smaller cohorts of open-source AID users of all age groups, including children and adolescents whose caregivers build and maintain these systems on their behalf [11-15]. Further studies reported improved sleep quality and uninterrupted sleep, in particular, reduced burden of diabetes management, increased confidence in achieving diabetes management goals, increased energy, and reduced mood swings among open-source AID users [15]. An in-silico study of the AndroidAPS algorithm showed similar glycemic improvements and concluded that this algorithm is both safe and effective [16].

Despite the potential benefits of open-source AID systems, little is known about the reasons why people with diabetes initially chose to use this technology. It is important to determine the lessons to be learned from the #WeAreNotWaiting movement, especially for stakeholders involved in research and commercial product development and regulation, such as academia, industry, health care professionals, governance, and regulatory bodies.

### Objectives

As part of the OPEN (Outcomes of Patients’ Evidence with Novel, Do-it-Yourself Artificial Pancreas Technology) Project, the aim of this study is to investigate motivational factors for building, using, and maintaining an open-source AID system among adults with diabetes (type 1, 2, and others) and caregivers of children and adolescents with diabetes, as well as their self-reported clinical outcomes, through a population-based survey [17].

## Methods

### Study Design and Participants

From November 2018 to March 2019, we conducted a web-based, cross-sectional survey titled *DIWHY* (Multimedia Appendices 1 and 2). The survey design was created by the patient-led OPEN consortium [17], in collaboration with open-source AID users, and piloted by a small number of them before the final release. The Checklist for Reporting Results of Internet E-Surveys was used to guide survey development [18]. The survey was approved by the Charité–Universitätsmedizin Berlin Ethics Committee (EA2/140/18). Participants were eligible if they were adults (aged >18 years), living with diabetes (type 1, 2, or other), or being caregivers of a child or an adolescent with diabetes using an open-source AID system.

### Procedures

Participants were invited through public announcements on the OPEN Project website, in the Facebook groups *Looped* (>6000 members) and *AndroidAPS users* (>1800 members, November 2018), other regional subgroups on Facebook, and by public posts on Twitter using the hashtags #WeAreNotWaiting and #DIYAPS. All posts were organic, meaning there was no paid promotion or targeted advertising of posts on any platform. All participants gave their consent electronically. Participation was anonymous and voluntary; no financial or other compensation was provided. Participants were able to choose between 2 language options (English and German). There was a version for adults with diabetes and one for caregivers. Data were collected and managed using secure Research Electronic Data Capture electronic data capture tools hosted at Charité [19].

### Measures

Initial questions focused on demographics, the type of open-source AID systems used, estimated commencement date, and 3 HbA_1c_ values each preinitiation and postinitiation of open-source AID (self-reported for adults; for caregivers, their child’s). In addition, participants were asked to provide their or their child’s average time in range (TIR; sensor glucose 70 mg/dL/4.0 mmol/L-180 mg/dL/10.0 mmol/L) before and after the commencement of open-source AID.

Subsequently, participants’ motivation to build an open-source AID was assessed with a single question: “What motivated you to build a Do-It-Yourself Artificial Pancreas system for yourself? Indicate your level of agreement with each statement*.*” A total of 14 fixed-choice statements followed to conclude the stem “I built a DIYAPS...” (eg, “...to achieve better glycemic control,” “...to improve my own sleep quality”). For each statement, a 5-point Likert-type scale was used (*fully applies* to *does not apply at all*). In addition, participants could indicate further motivational factors using free text.

### Quantitative Analysis and Statistical Testing

To ensure the reporting of robust parameters regarding HbA_1c_ levels, entries with more than one missing HbA_1c_ value either before or after open-source AID implementation were not considered in the calculation of arithmetic means, SDs, and statistical tests related to HbA_1c_. The reduction in the average HbA_1c_ levels before and after open-source AID implementation was assessed using the Wilcoxon signed-rank test (*P* value threshold of .05, paired: *TRUE*, and alternative hypothesis: *greater*). Entries not providing TIR values before and after open-source AID implementation were not considered for the computation of TIR-related descriptive statistics and testing for the increased TIR after open-source AID implementation (same statistical test as for HbA_1c_, with alternative hypothesis set to *lower*). Quantitative analyses were conducted within the R programming framework (v4.0.2; R Core Team), and the ggplot2 package was used to generate figures.

### Content Analysis

Content analysis was performed to analyze responses to open-ended questions [20]. A total of 3 researchers coded data and analyzed the responses thematically in 2 rounds. After the first round, which was open, inductive, and independent, 3 lists of codes were merged and combined into a final version. The second round of coding was deductive, and each of the coders assessed the content according to the final list of codes. The interrater reliability (percentage agreement for multiple raters) method was used to calculate the level of agreement between coders, and the final list of the most frequently discussed codes was generated [21]. Codes were then compared with assess the level of similarity, for example, an interrater reliability result of 100% indicated that all codes generated by individual coders matched.

## Results

### Characteristics of the Study Cohort

A total of 1125 individuals participated in the *DIWHY* survey. After excluding 25.6% (288/1125) incomplete responses, data from 897 individuals over 35 countries were analyzed. Detailed demographic characteristics are shown in Table 1. Participants were mostly from Europe (691/897, 77%), whereas 14% (125/897) were from North America, and 9% (78/897) were from other continents. Most adults (599/722, 82.9%) and caregivers (153/175, 87.4%) had a university degree or higher. Of the respondents, 26% (236/897) had a professional background in information technology and 19% (170/897) in biomedicine or health care. Furthermore, 82% (736/897) of the participants reported out-of-pocket expenses, with an average of US $530 and a maximum of US $1000 per year. In both groups, various types of open-source AID systems were used regularly, with Loop being the most popular system in North America and AndroidAPS being the most frequently used system in Europe. Otherwise, the geographical location and household income did not indicate any specific patterns.

**Table 1:** Participants’ demographic and self-reported clinical characteristics.

Participant demographics						Children and adolescents (n=175)			Adults (n=722)			Total (N=897)
**People with diabetes, gender, n (%)**												
			Female		83 (47.4)			311 (43)			394 (43.8)	
			Male		92 (52.6)			411 (56.8)			503 (55.9)	
			Other		0 (0)			2 (0.3)			2 (0.2)	
People with diabetes, average age, years (SD)						9.7 (4.0)			41.8 (11.8)			35.6 (16.7)
**Type of diabetes, n (%)**												
			Type 1		174 (99.4)			714 (98.9)			888 (98.9)	
			Type 2		0 (0)			4 (0.6)			4 (0.4)	
			Other		1 (0.6)			4 (0.6)			5 (0.6)	
Average duration of diabetes, years (SD)						5.1 (3.9)			25.2 (13.3)			21.4 (14.4)
Average duration of open-source AID^a^ use, mean (SD)						10.3 (10.0)			10.0 (19.1)			10.1 (17.6)
**Type of open-source AID used regularly, n (%)**												
			OpenAPS		42 (28.4)			104 (16.6)			146 (18.8)	
			AndroidAPS		71 (48)			380 (60.6)			451 (58.2)	
			Loop		42 (28.4)			179 (28.5)			221 (28.5)	
			Other^b^		5 (3.4)			39 (5)			44 (5.7)	
**Region, country of residence, n (%)**												
	**Europe**				130 (74.3)			561 (77.6)			691 (76.9)	
		Austria		3 (1.7)			23 (3.2)			26 (2.9)		
		Bulgaria		9 (5.1)			7 (1)			16 (1.8)		
		Czech Republic		12 (6.9)			9 (1.2)			21 (2.3)		
		Finland		8 (4.6)			10 (1.4)			18 (2)		
		Germany		46 (26.3)			363 (50.2)			409 (45.5)		
		The Netherlands		0 (0)			10 (1.4)			10 (1.1)		
		Spain		3 (1.7)			11 (1.5)			14 (1.6)		
		Sweden		8 (4.6)			3 (0.4)			11 (1.2)		
		The United Kingdom		23 (13.1)			99 (13.7)			122 (13.6)		
		Other^c^		14 (8)			35 (4.8)			49 (5.5)		
	**North America**				21 (12)			104 (13.9)			125 (13.4)	
		Canada		5 (2.9)			18 (2.5)			23 (2.6)		
		The United States		16 (9.1)			86 (11.9)			102 (11.3)		
	**Asia**				12 (6.9)			14 (2.9)			26 (2.9)	
		South Korea		12 (6.9)			10 (1.4)			22 (2.4)		
		Others^d^		0 (0)			4 (0.4)			4 (0.4)		
	**Western Pacific**				12 (6.9)			39 (5.4)			51 (5.7)	
		Australia		12 (6.9)			29 (4)			41 (4.5)		
		New Zealand		0 (0)			10 (1.4)			10 (1.1)		
	**Africa**				0 (0)			1 (0.1)			1 (0.1)	
		South Africa		0 (0)			1 (0.1)			1 (0.1)		
**Education: highest completed, n (%)**												
			No or some high school		19 (10.9)			54 (7.6)			73 (8.1)	
			High school		16 (9.2)			67 (9.4)			58 (6.5)	
			University		111 (64.1)			449 (62.9)			627 (71.1)	
			Degree or diploma		21 (12.1)			61 (8.5)			82 (9.2)	
			Doctorate		21 (12.1)			89 (12.4)			110 (12.4)	
**Occupational status^e^, n (%)**												
			Full time		101 (58.4)			486 (67.6)			587 (65.8)	
			Part time		55 (31.8)			114 (15.9)			169 (18.9)	
			Unemployed		10 (5.8)			6 (0.8)			16 (1.8)	
			Retired		0 (0)			38 (5.3)			38 (4.3)	
			Student		2 (1.2)			58 (8.1)			60 (6.7)	
			Other		5 (2.9)			17 (2.4)			22 (2.4)	
**Professional background^e^, n (%)**												
			Medicine		24 (18.5)			102 (19.5)			126 (19.2)	
			Tech		35 (26.9)			137 (26.2)			172 (26.3)	
			Other		71 (54.6)			284 (54.3)			355 (54.4)	
**Household annual net income^e^,** **US $, n (%)**												
			<20,000		19 (12)			87 (14.1)			106 (13.6)	
			20,000 to 34,999		12 (7.6)			60 (9.7)			72 (9.2)	
			35,000 to 49,999		19 (12)			88 (14.2)			107 (13.7)	
			50,000 to 74,999		33 (20.9)			138 (22.3)			171 (22.1)	
			75,000 to 99,999		24 (15.2)			84 (13.6)			108 (13.9)	
			>100,000		40 (25.9)			124 (20)			165 (21.2)	

^a^AID: automated insulin delivery.

^b^xDrip, Nightscout, offline uploader for Medtronic 600 series, HAPP, and custom or own developments.

^c^Belgium, Croatia, Denmark, France, Greece, Hungary, Ireland, Italy, Lithuania, Luxembourg, Norway, Poland, Portugal, Russia, Slovakia, and Switzerland.

^d^Hong Kong, Kuwait, Palestine, and Singapore.

^e^For adults: own; for caregivers: caregivers.

### Motivations to Commence Open-source AID Use

**Figure 1:**
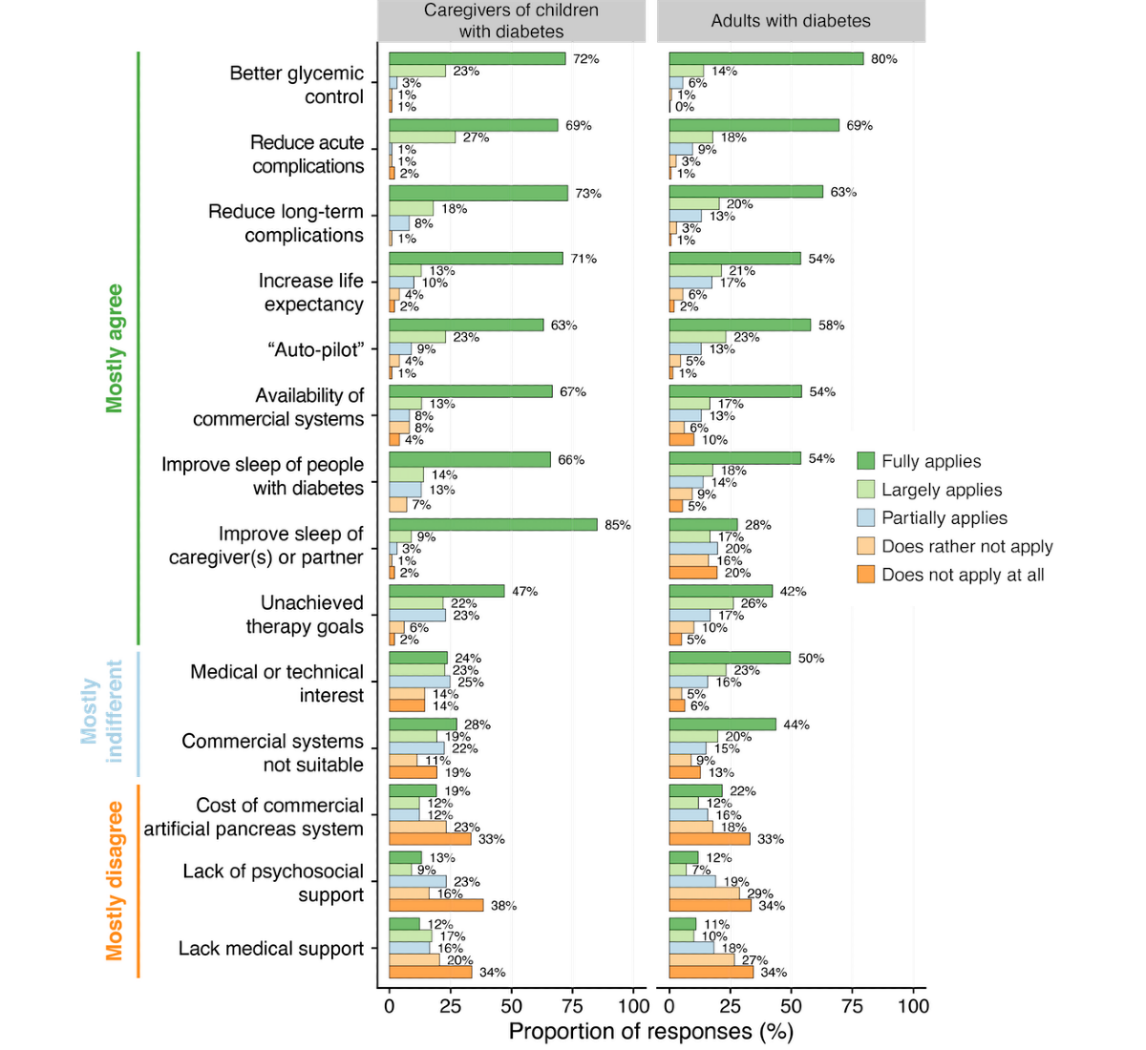
Motivations for building an open-source automated insulin delivery system. The x-axis shows the percentage of responses for each motivation question (y-axis). Bar colors represent the degree of relevance ranging from “does not apply at all” to “fully applies.” The left and right columns show the responses of caregivers of children with diabetes and adults with diabetes, respectively. Responses are ranked from the most frequently endorsed motivations (top) to the less frequently endorsed (bottom).

As shown in Figure 1, the most frequently endorsed motivations of adults as well as caregivers (as *fully applies* or *largely applies*) were to improve the overall glycemic control (476/509 adults, 93.5%; 95/100 caregivers, 95%), reduce the risk of acute (443/508 adults, 87.2%; 96/100 caregivers, 96%) and long-term complications (421/505 adults, 83.4%; 91/100 caregivers, 91%), put diabetes on *auto-pilot* mode and interact less frequently with diabetes technology (413/509 adults, 81.1%; 86/100 caregivers, 86%), increase their own or their child’s life expectancy (381/507 adults, 75.1%; 84/100 caregivers, 84%), and improve their own or their child’s sleep quality (364/508, adults 71.7%; 80/100 caregivers; 80%), because of the lack of commercially available closed-loop systems in their countries (359/507 adults, 70.8%; 79/99 caregivers, 80%) and unachieved therapy goals with the therapy options available to them (348/509 adults, 68.4%; 69/100 caregivers, 69%).

Overall, the motivations of adults and caregivers of children and adolescents with diabetes were largely similar. As the most noticeable difference between the 2 groups, improvement in their own sleep quality (94/100, 94%) was a stronger motivation for caregivers compared with adults with respect to their partners or families (225/505, 44.6%). Curiosity (medical or technical interest) was endorsed more frequently by adults with diabetes (367/503, 73.0%) than by caregivers (45/97, 47%). Some believed that commercial systems did not suit their own or their child’s individual needs, more frequently reported by adults (316/498, 63.5%) than by caregivers (46/98, 47%). Out-of-pocket costs related to the use of commercially available systems (166/496 adults, 33.5%; 31/99 caregivers, 31%) played a subordinate role. Lack of adequate medical support (105/501 adults, 21.0%; 29/98 caregivers, 30%) or psychosocial support (94/501 adults, 19.0%; 22/99 caregivers, 22%) were less frequently endorsed as motivating factors, although caregivers more frequently indicated a lack of medical support.

### Further Motivations

In addition to the 14 predefined items, participants could indicate further motivation in an open-text field. In total, 127 participants (103 adults and 24 caregivers) provided a free-text response. Textbox 1 provides a list, as well as representative quotes, of the respondents. In the independent coders’ selection of first-choice codes, there was an 83% interrater agreement between them (Multimedia Appendix 3).

**Illustrative quotes from adults with diabetes and the caregivers of children or adolescents with diabetes, highlighting additional motivation factors to build an open-source automated insulin delivery system.**

**Improving Diabetes Management**

The psychological benefits of being able to significantly improve active control over diabetes and outcomes, rather than being more passively subjected to it.

Another important reason for me is that I FINALLY have an overview of all data combined for later analysis but also direct decisions (values instead of opinions).

**Improving Quality of Life or Reducing the Burden of Diabetes Management**

I chose DIY to decrease the demands of living with diabetes every day, around the clock. I also needed help consistently combatting the dawn phenomena, where I would wake up either too high, or too low from overcorrecting.

His quality of life (staying with friends, knowing we can remotely monitor and assist, knowing that loop will help correct if he makes a mistake, attending sports training independently) is vastly improved. We can sleep! A happier, healthier family.

Freedom to participate in normal 8 year old life eg play dates without having to pre-plan everything.

Management of diabetes is helped by support but it is very much a self managed disease and requires 24/7 attention. Closed looping makes it just so much better, much of the time I can leave AAPS to take care of basals by itself. Quality of life is so much better. I can sleep without worrying about not waking up because of a bad hypo.

We only wanted the best for our son. He should get exactly the same chances in life as his friends/children of the same age.

To improve constant feeling of failure.

**Diabetes Distress or Burnout**

Tired of diabetes after almost 30 years [...] The first real relief for me in my everyday life as a single mom.

To reduce psychological distress, to be able to take responsibility for the course of diabetes, to enjoy life more since you are not torpedoed by Hypos and Hypers. Freedom despite technically higher dependency.

There was no other way. The available treatments just did not control my diabetes sufficiently. The pressure and hopelessness of that scenario caused major mental health problems.

I’ve lived my whole life [like] this & can’t take it anymore. Too hard to do. Worst problem is “brain fog” & lack of energy due to blood sugar swings & hypoglycemic unawareness. I carry guilt for causing my family to lose sleep & carry the burden of diabetes [...]. [The] burden of diabetes is terrible.

**Autonomy**

I feel so empowered by building my own system and taking control of my T1D. It’s an awesome feeling!

Daughter has learning difficulties, to make life easier for her and be less dependent on support, which in turn allows her to live a more independent life.

To regain a sense of control on my diabetes management. I felt I was becoming dependent on my specialist for interpreting the adjustments needed for my insulin regime.

Independent sleepovers with friends (without parents).

To expand our daughter’s independence and make her therapy decisions easier.

**Dissatisfaction With Available Technology, Choice and Health Care**

Out of frustration with the existing designs seeming to have prioritized all stakeholders other than patients.

Commercial closed loop systems do not allow users to specify a custom target BG but instead hard wire an unambitious target more concerned with legal liability that doesn’t respect the autonomy, needs and wishes of the user.

Dissatisfied with commercially available options and choice in the market space. No other option is appealing or provides the level of control and true artificial pancreas functions OR user interface.

Doctors and hospitals have been telling me for years that things are simply fluctuating for me (hormones, stress, sensitivity to movement) and that you can’t do anything about it. ‘Resistant to all treatment options’ and well-educated. Unfortunately with no success.

We were desperate for something to use all the CGM data without sending our child crazy with in[sistent] requests for the pump to set low temps etc. We were infuriated by the business based decisions around closed loop in Australia - only the 640G was available and it was the worst decision for management and burden that we ever made. Now (as in within this fortnight) the 670G is available but still, no one can get training or sensors. We have been looping for nearly 3 years. If we hadn’t then we would still be waiting today.

**Improving Sleep Quality**

Sleep was the main reason followed by time in range. However, after all these years I still wake up but go back to sleep quickly.

Frequently woke up from sensor alarms, make corrections and still wake up in the morning with a high or low glucose. Since closed looping, I get into bed knowing that Loop will keep me in range and I will wake up with a neat glucose. The only alarms I would ever get during the first period of closed looping were compression lows, and with the experience of loop keeping me in range I am now even confident enough to shut down all CGM alerts. Makes a huge difference for both me and boyfriend now that we start our days well rested. Every single day.

**Safety or Reducing Severe Hypoglycemia**

Too many overnight hypos that require help.

My child was overdosed on insulin twice by untrained teacher aides at school and if it was not for DIY looping technology- I would not have known about this at all until too late. Seeing the boluses appear on nightscout on real-time allowed me to question the dose and sugar treatment could commence preventatively than child actually going into severe hypo.

**“DIY mindset” or Early Adopter of Technology**

I was going to build my own and found existing projects.

Early adopter of all diabetes technologies. Turns disadvantage into a challenge.

I love tinkering and making things. I’d always rather DIY, in many aspects of life.

I initially built a closed DIY APS for a hackathon project out of pure tech curiosity. I planned to use the system for only 12 hours and then give a presentation to other employees at our company involved with the hackathon. After 12 hours, I realized I was never going to stop using it. Once on the system, almost every single one of the survey questions above are a “Fully Applies” as to why I decided to stay on the DIY APS.

I’m a doctor and I’d like to test the closed loop first for myself and then use it in the future in my patients’ treatment.

**Community Spirit**

Being part of the community of selfless, generous, caring, and talented people willing to volunteer their time, knowledge, skills and experience to the benefit of the community.

Something that also influenced me to move to a DIY system was the support from the community, and the general feeling that the community gives. It feel like I am part of a big people- powered movement. It feels like a revolution.

I felt a strong moral and ethical imperative that technology should serve people.

Help others to have healthier life.

**Comorbidities**

I started on AndroidAPS when I was diagnosed with cancer needing chemotherapy. I found it extremely beneficial especially for those times when I was at my lowest and unable to control my BGs in the old way because of insulin resistance. Also when I was admitted to hospital because of infections and sepsis it was a godsend.

More beneficial sexual activity, PDE-5 inhibitors no longer required.

Because of other conditions, I have to take cortisone in different doses on a regular basis. This has made my diabetes management so difficult. The loop absorbs my BG fluctuations much better.

Achalasia (food gets stuck in the esophagus at night), making blood sugar uncontrollable.

I have been on a pump since 1992. I was on the 670G for over a year, and I felt helpless in my efforts to achieve excellent glycemic control while still living my random and not standardized life, where I eat when I am hungry, or forget to eat, and where pre-bolusing is dangerous, because I also have ADHD and I have forgotten to eat many times. My insulin needs vary depending on what I do in terms of activity, but also randomly on the day of the week, the time of the month and many other factors that i don’t understand. On the 670G every weekend of high physical activity was followed by a couple of days of high BGs due to the user’s inability to interact with the proprietary algorithms (Oh I am so done with Medtronic now).

**Diabetes-Related Complications**

After 29 years of MDI and [...] retinopathy I decided to improve my health. I’ve researched several ways to improve control. Ultimately autonomy is the box I needed ticked! AAPS ticks that box 100%.

Gastroparesis, I barely had nights where I wasn’t over 200 half the night. With the G5 I was woken up at 170 and was able to intervene. Since the loop and some completed goals, I fall asleep again because the loop prevents the uncontrollable rise!

Heart operation after 30+ years of poorly controlled diabetes.

**Female Health**

Wanted better control for pregnancy.

As someone whose hormone levels are not considered standard and rapidly change, the ability to [have] a helping hand to smooth out these Diabetes related complications (notably hyperglycemia episodes) was very important to me, as the situation is never the same twice and requires different treatment on a day-to-day basis.

Deteriorating HbA_1c_ due to puberty and insulin resistance. Massive amounts of insulin needed giving unpredictable blood glucose.

After manifestation of T1D, we made very high demands on HbA_1c_ and TiR for the benefit of our daughter...but with the onset of puberty, this led to an almost impossible workload (correcting 10-15 times at night).

To have more insight as to why my blood glucose was so volatile due to changing hormones (menopause).

**Out-of-Pocket Expenses**

It was questionable whether I would meet the health insurer’s criteria for the Minimed 670 system for reimbursement. I don’t have a CGM either, just the Freestyle Libre with an additional transmitter.

**Improving Performance**

To improve my work at the office.

To improve athletic performance by controlling night time blood sugars.

**Curiosity**

To learn more about my diabetes in general. You have to acquire a lot of knowledge (technical as well as physiological aspects) before you start looping, and you get excellent support from developers and the community.

The fact alone that you can be curious again about something new to the diabetes field, to see a form of therapy as an exciting challenge, plus the (so far not yet fulfilled hope) to finally better control the hardly controllable variating [postprandial] values.

Most of the indicated *other motivations* provided greater details about the 14 predefined statements. The most frequently mentioned motivations for all—adults and caregivers—were *better management* and *reducing the disease burden*. The first motivation appears consistent with several statements related to hypoglycemia and hyperglycemia and risk reduction, whereas the second motivation may correspond with *to put diabetes management more on auto-pilot and interact less frequently with the therapy system*. This aspect and sleep quality are understood as the quality of life gains. Of motivations not covered by the predefined responses, the most frequently mentioned was *autonomy gain* in both adults and children or adolescents, as indicated by the caregivers. All these aspects were associated with improvements in family life:

This is for my wife. She wants me to live forever, this is as close as I can do for her.

Psychosocial aspects, ranging from diabetes burnout and distress to a desire to improve athletic performance to increasing efficacy at work, were also identified as important motivating factors. The following comments illustrate the wide-ranging benefits experienced by many participants after adopting the technology:

Management of diabetes is helped by support, but it is very much a self-managed disease and requires 24/7 attention. Closed looping makes it just so much better, much of the time I can leave [AndroidAPS] to take care of basals by itself. Quality of life is so much better. I can sleep without worrying about not waking up because of a bad hypo. [...] I am so grateful to all the software developers who have freely given their expertise and time to make this possible.

As highlighted in the example above, an important role was played by the *community spirit* and peer support in social networks:

Something that also influenced me [...] was the support from the community, and the general feeling that the community gives. It feels like I am part of a big people-powered movement. It feels like a revolution.

Not only a *Do-It-Yourself* mindset and being *early adopters* of technology but also being motivated and empowered to improve one’s life were frequently mentioned:

Because it’s the most natural thing to do, after getting to know that it’s possible. Because I could.

Some motivations included other health-related aspects such as improving the management of existing diabetes-related complications and increasing safety by avoiding severe hypoglycemia. Other comorbidities, such as cancer, sexual health difficulties, or conditions requiring cortisone treatment, were also mentioned:

*I have to take cortisone in different doses on a regular basis. This has made my diabetes management so difficult. The loop absorbs my BG fluctuations much better.*

Women and caregivers of female children highlighted female health aspects such as hormone-related changes in insulin sensitivity, family planning, and pregnancy as reasons to commence open-source AID:

To have more insight as to why my BG was so volatile due to changing hormones (menopause).

For some, special features were only offered by open-source AID and not by commercial systems, that is, customizable targets and the option to bolus from a smartwatch. For caregivers, remote real-time access to their child’s data and the option to remotely control their child’s AID system have been frequently described.

### Improved Glycemic Outcomes Across All Age Groups and Genders

**Figure 2:**
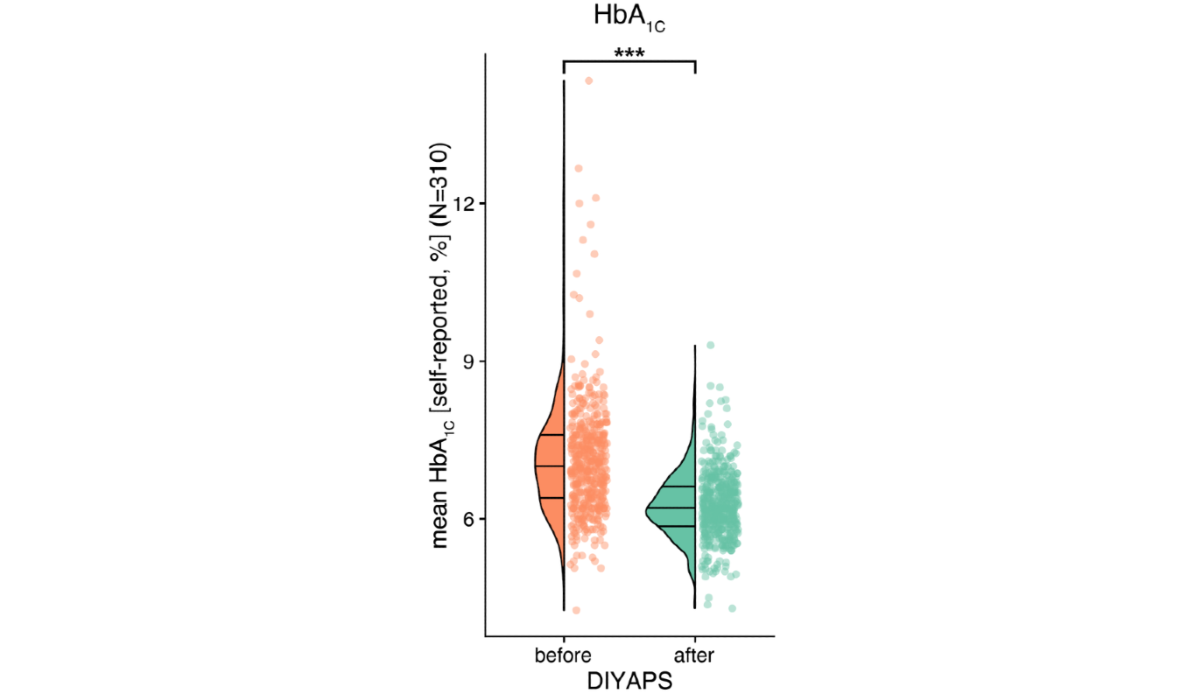
Positive effects of open-source automated insulin delivery on clinical outcomes: average self-reported glycated hemoglobin (%; y-axis) for all 310 respondents, before and after open-source automated insulin delivery (x-axis) distinguished by orange and green colors, respectively. The left side is displayed as a density plot, with horizontal lines indicating quartiles. The right side depicts the data as a scatter plot. DIYAPS: Do-it-Yourself Artificial Pancreas System; HbA_1c_: glycated hemoglobin.

To assess glycemic outcomes, participants were asked to report their or their child’s 3 most recent HbA_1c_ results before as well as the first 3 HbA_1c_ results after commencing the open-source AID. HbA_1c_ levels decreased significantly following open-source AID implementation (*P*<.001) from an average of 7.14% (SD 1.13%; mean 54.5 mmol/mol, SD 12.4) to 6.24% (SD 0.64%; mean 44.7 mmol/mol, SD 7.0), with an effect size of −0.9% (Figure 2).

**Figure 3:**
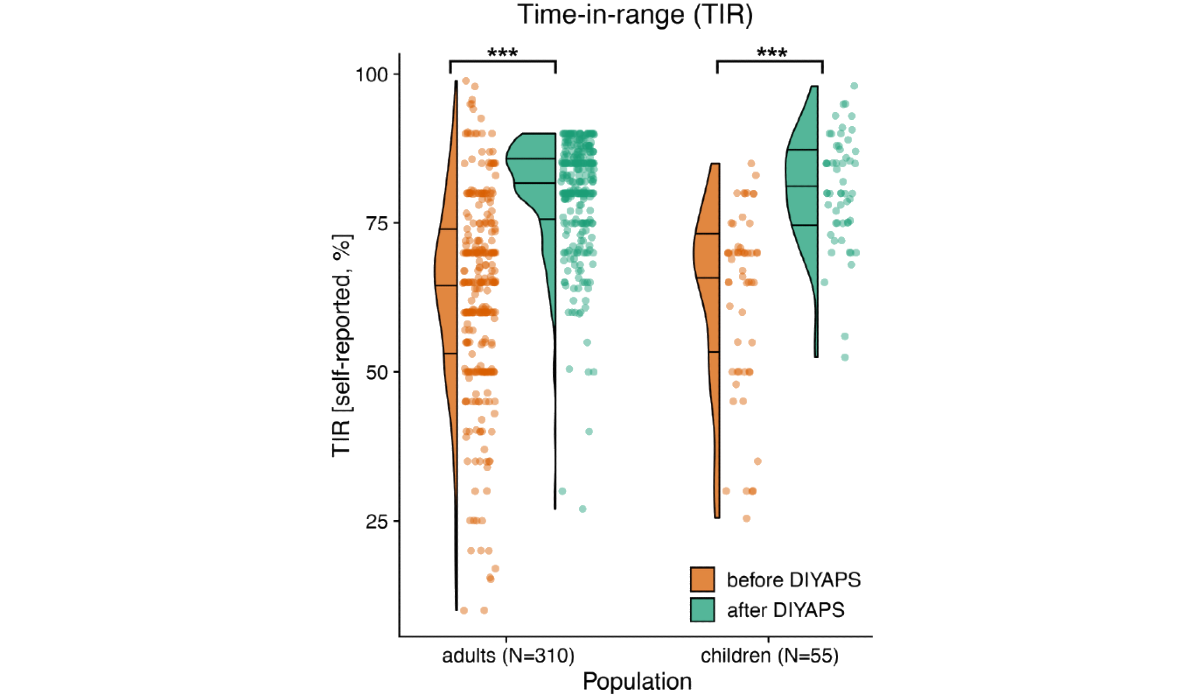
Self-reported time in range (%; x-axis) of adults and children with diabetes, before and after implementing an open-source automated insulin delivery system. The left side is displayed as a density plot, with horizontal lines indicating quartiles. The right side depicts the data as a scatter plot.

**Figure 4:**
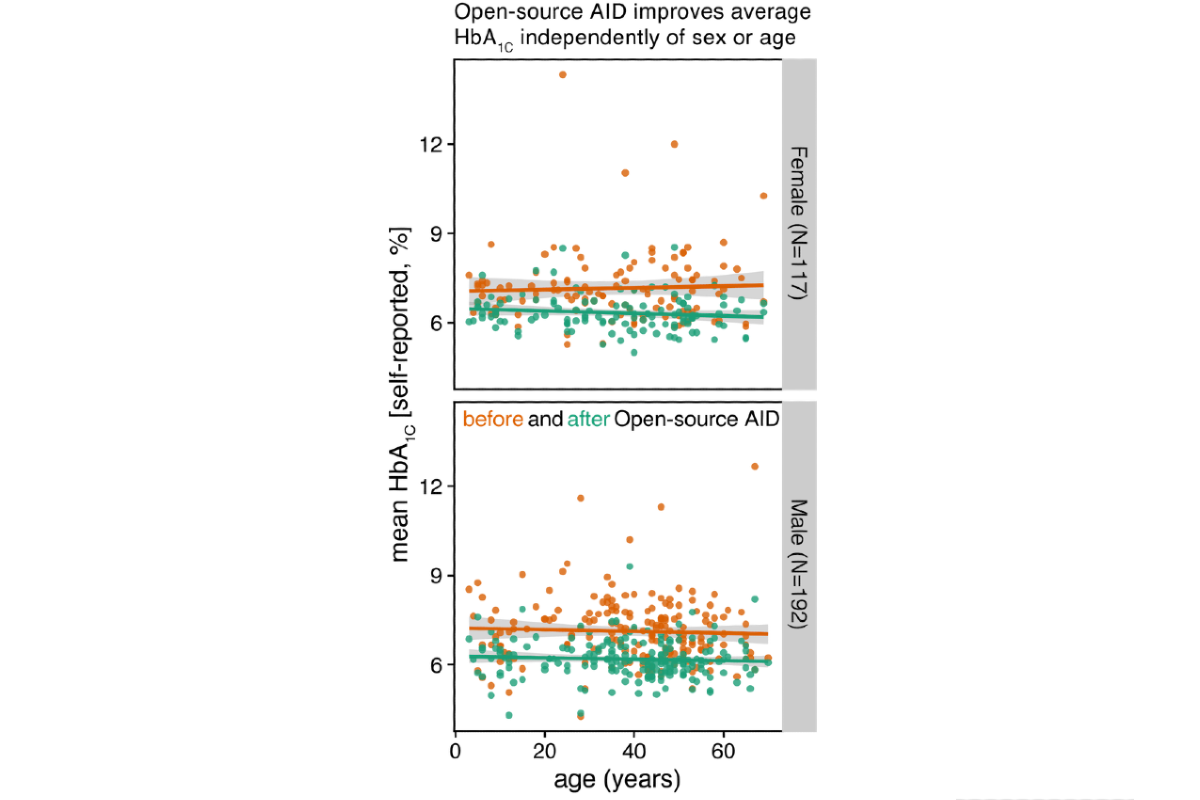
Improvements in self-reported glycated hemoglobin levels associated with open-source automated insulin delivery, independent of age or gender: relation between average glycated hemoglobin levels (%; y-axis) and age (x-axis), shown separately for female and male respondents (top and bottom rows, respectively). Colors separate average glycated levels before (orange) and after (green) open-source automated insulin delivery implementation. Each point represents one respondent after filtering of responses (the Methods section). Solid lines and their gray areas represent the trend and standard error for the respective groups. AID: automated insulin delivery; HbA_1c_: glycated hemoglobin.

**Figure 5:**
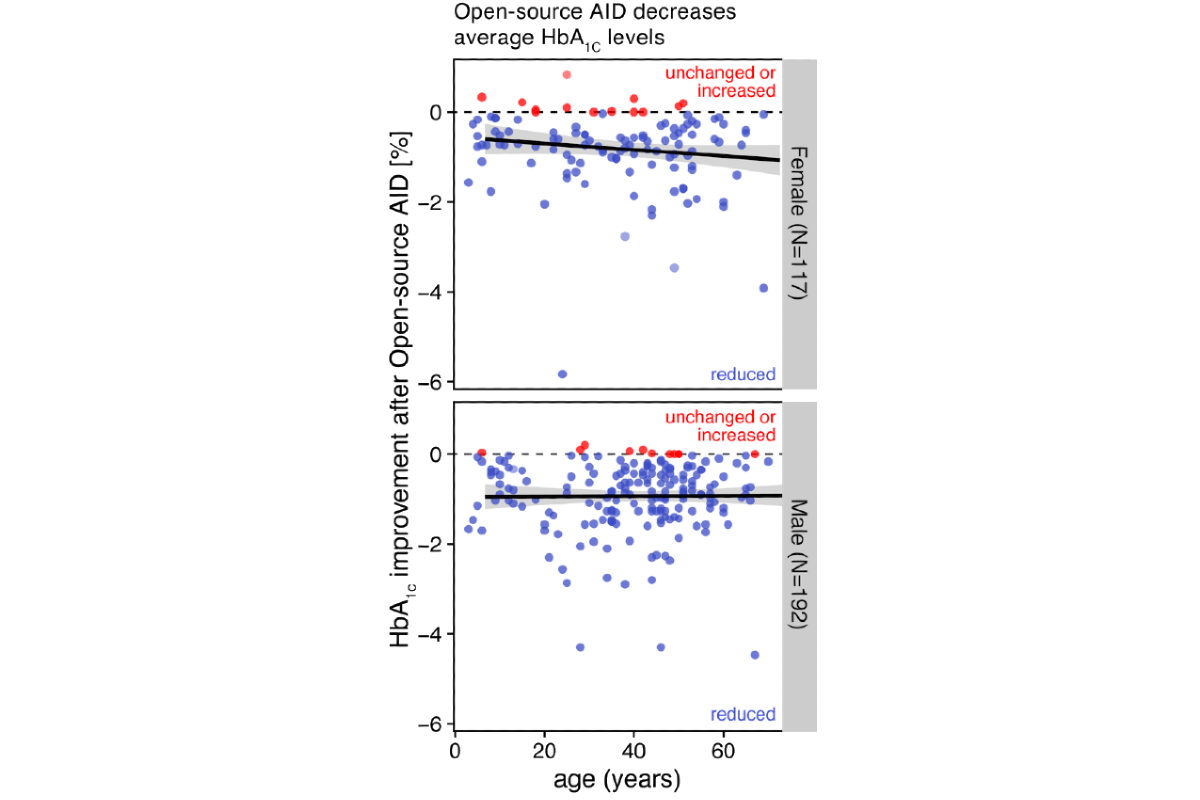
Improvements in self-reported glycated hemoglobin levels associated with open-source automated insulin delivery, independent of age or gender: the y-axis shows the difference of average glycated hemoglobin levels after open-source automated insulin delivery, compared with before its implementation. Colors distinguish respondents with reduced average glycated hemoglobin (blue) from those with unchanged or increased glycated hemoglobin (red). AID: automated insulin delivery; HbA_1c_: glycated hemoglobin.

The average self-reported TIR across adults and children with diabetes significantly increased by +17.4%, from 62.96% (SD 16.18%) to 80.34% (SD 9.41%; *P*<.001; Figure 3). Similar outcomes were observed separately for adults and children with diabetes (Multimedia Appendix 3) and were independent of age and sex (Figure 4). Overall, 92.3% (286/310) of the respondents reported a decreased average HbA_1c_ level (Figure 5).

## Discussion

### Principal Findings

This study is the first to systematically analyze the motivations found within the #WeAreNotWaiting movement of people with diabetes, who have built and maintained their open-source AID systems and created their own ecosystem of international self-support networks. To the best of the authors’ knowledge, this is also the largest study reporting the self-reported clinical outcomes of open-source AID users across several continents. We found large effect sizes for self-reported improvements in HbA_1c_ (−0.9% on average) and TIR (+17.4% on average), indicating considerable biomedical benefits associated with open-source AID, which were independent of sex and age.

### Why #WeAreNotWaiting: Main Motivators to Choose an Open-source AID

The main motivators for adults were improvements in overall glycemic and long-term outcomes and quality of life, whereas the strongest motivation for caregivers was improvement of their own sleep, followed by improved glycemia of the child and possibility of remotely controlling glycemia and insulin delivery via the internet. The results indicate that motivations are configured differently among caregivers and that other motivations also scored a high level of consensus among the respondents. These findings suggest that motivation to transition toward open-source AID is multifaceted and complex, with reasoning and decision making bound up with the psychological and social intricacies of individuals’ lives.

### Improvement in Sleep Quality

Caregivers experience reduced sleep quality because of fear of hypoglycemia, which often requires them to regularly check their child’s glucose levels overnight [22]. In our study, caregivers reported experiencing fewer demands and less apprehensiveness regarding their child’s glucose levels at nighttime. As shown in the free-text responses, open-source AID also appears to offer caregivers with the reassurance necessary to provide their child more autonomy and engage in activities that might otherwise present a risk, such as having *a sleepover* with friends. Previous studies in adults using open-source AID have shown self-reported improvement in sleep quality [23,24]. These initial findings indicate a substantial benefit for users and caregivers for sleep and most likely for their psychological and physical well-being. Poor quality of sleep negatively affects the psychological well-being, cognitive functioning, and a diverse range of hormones that affect the regulation of appetite and our homeostatic systems as well as the immune system [25-34]. Recent research also points to sleep as impacting the actual maintenance of the brain and our DNA regenerative systems [35]. Thus, AID may play an important role in improving the psychological and physical health of people with diabetes and their family members. However, it has been noted elsewhere that the potential discomfort or inconvenience of wearing devices and overnight alarms may also hamper the benefits for some users [36].

### The Importance of Customizability: One Size Does Not Fit All

The majority of participants reported that currently approved and available commercial therapy options may not be sufficiently flexible or customizable to fulfill their or their children’s individual needs. Among caregivers, features only available in open-source AID, in particular, the possibility of remote management was the main additional motivation. A wider range of features and adjustable settings to improve user experience may be beneficial for people with diabetes of all ages, which mirrors a recent study in very young children using a commercial AID [36]. Interestingly, for many adult respondents, *curiosity* was cited as an important motivation. In contrast, curiosity was a much lower motivating factor for caregivers who chose to build a system for more practical or psychosocial reasons.

### Do-it-Yourself Is Not Do-it-Alone: The Impact of Peer Support

The ability to receive and provide support within the do-it-yourself (DIY) community and observe the success of others was an important motivating factor associated with opting to use open-source AID for some. Obtaining and exchanging information and advice from open forums limits the spread of misinformation because other users constitute a community of inquirers ready to challenge and correct spurious or misleading information [37]. Although open-source AID is individualized and patient focused, it is also a grassroots community-driven movement. The number of responses to our survey reflects the enthusiasm and importance of open-source AID. In challenging traditional top-down hierarchies in medicine, open-source AID presents a patient-focused initiative that serves to empower people with diabetes through personalized technology. Because of the availability of current technology and individualized innovations, open-source AID has previously been described as having the potential to democratize health care, revolutionizing treatment and the way people with diabetes as well as other stakeholders such as care teams, researchers, and device manufacturers view chronic conditions such as diabetes [38]. The importance of peer support in the context of open-source AID use has recently been highlighted elsewhere, and a sense of community underpinning the development and diffusion of open-source AID has been emphasized by individual users [39]. Further research should consider community phenomena as an integral part of the DIY experience.

### Strengths and Limitations

This study is the first to investigate motivations of users and caregivers to build and use open-source AID. In addition, this is the largest study that reports self-reported clinical outcomes of open-source AID users globally and adds to the existing evidence base around glycemic outcomes in smaller cohorts [11-15]. At the time of data collection, it surveyed the majority of open-source AID users worldwide, with 897 respondents of a population estimated in 2018 (N=1500). The sample is impressive not only in size but also because people with diabetes from various continents and regions of the world are represented. Of other strengths, this study has been conducted by an interdisciplinary consortium, with members of the diabetes community directly involved. However, this firsthand experience should be acknowledged as a potential bias. In addition, a key limitation of the study is the fact that self-reported outcomes have not been corroborated by clinical records. Some may consider this has potential for inaccuracy, that is, by lacking precision as witnessed by the overaccumulation of rounded TIR values or to be biased by the specificity of the population that participated. However, other studies have found that real-world data are robust and reliable [40]. We acknowledge that open-source AID users constitute a specific group of people with diabetes who may be highly motivated, engaged, and willing to improve the quality of their diabetes care and life, limiting the scope of our findings to this group. However, a recent study of newly available commercial AID systems indicates that users are similarly motivated to achieve the best possible outcomes but are dissatisfied with postprandial glycemic outcomes under commercial AID systems [41]. The earliest adopters of available commercial AID technology may be more similar to the group of people with diabetes choosing open-source AID in terms of motivation and engagement than expected. The lack of widespread availability of AID technology in general, including commercial systems with regulatory approval, at the time of the study likely influenced the perspectives of people with diabetes choosing open-source systems. In the future, wider availability—and, importantly, better funding or insurance coverage of commercial AID systems—may further influence this cohort. Similarly, it remains to be seen if the predicted *second generation* of commercial systems—with a hypothesis of increased sophistication or improvements on the first-generation devices and algorithms—will enable people with diabetes to achieve results similar to those they are currently achieving with their chosen open-source AID system.

It should also be noted that those who benefit from and continue to use open-source AID may be motivated to share their positive experiences. Although the survey was open to people with type 2 and gestational diabetes, it was completed almost exclusively by adults and caregivers of children with type 1 diabetes. This is likely a reasonable reflection of the DIY community, but efforts need to be made in the future to encourage participation of those with other types of diabetes. The high percentage of respondents from Europe may be influenced by the fact that the majority of the research team is EU based, which may be another bias. This may also be explained by the characteristics of the European health services provision and reimbursement of diabetes-related technology, which may provide a greater degree of accessibility of the underlying components needed (eg, pumps and continuous glucose monitors). Language barriers may have limited responses from other parts of the world as the survey was only available in 2 languages. Finally, the majority of participants had a university degree, suggesting that open-source AID uptake is more common among people of higher socioeconomic status. Increasing socioeconomic inequalities in access to the underlying technologies needed to build an open-source AID system may help to explain some of these variations. Thus, further investigation into how the wider diffusion of open-source AID is conditioned by factors such as social class, gender, age, and geographic location is required.

### Conclusions

This study provides new insights into the factors that motivate people to adopt *DIY* solutions in relation to their diabetes and beyond. Our findings contribute to a better understanding of the unmet needs of people with diabetes and some of the current challenges in the uptake of AID technology. This study, alongside other efforts in the DIY community space, can help key stakeholders, including academia, the medical device industry, regulators, health care providers, and care teams, to better address some of the fundamental gaps and needs that still exist for people with diabetes worldwide, even with the advent of first-generation commercial AID systems. The DIY movement has resulted in impactful solutions addressing the unmet needs of people with diabetes and represents an exemplary case of how informed and connected patients are shaping the direction of technological innovation in diabetes care and potentially for other areas of health care in the future.

## Appendix

Multimedia Appendix 1 Questionnaire for people with diabetes.

Multimedia Appendix 2 Questionnaire for caregivers of children and adolescents with diabetes.

Multimedia Appendix 3 Supplementary tables 1 (codes and frequency of mentioned additional motivations as responses to the open-ended question) and 2 (detail of time-in-range improvements following open-source AID implementation).

## Abbreviations

AID: automated insulin delivery
DIY: do-it-yourself
DIYAPS: Do-It-Yourself Artificial Pancreas Systems
HbA_1c_: glycated hemoglobin
OPEN: Outcomes of Patients’ Evidence with Novel, Do-it-Yourself Artificial Pancreas Technology
TIR: time in range
