# Supplementary material for: Why #WeAreNotWaiting—Motivations and Self-Reported Outcomes Among Users of Open-source Automated Insulin Delivery Systems: Multinational Survey
Source: J Med Internet Res. 2021 Jun 7;23(6):e25409. doi: 10.2196/25409 (PMC8218212; doi:10.2196/25409)
Supplement: Multimedia Appendix 2 [file jmir_v23i6e25409_app2.pdf]

DiWHY EN For Caregivers

Welcome to DIWHY!

We hereby offer you to participate in the study "DIWHY - Motivations, Barriers and Retention Factors Do-It-Yourself Artificial Pancreas System Users in Real-World Use". The following text is intended to explain the objectives of our investigation and how to take part in the survey. Please do not hesitate to contact our study team in case of any doubts and/or questions.

Purpose of the research: This study aims to examine the motivations of current Do-It-Yourself Artificial Pancreas users and to explore the barriers to, and facilitators of, building and maintaining such systems and how they might differ by socioeconomic status, ethnicity, gender and age.

Type of Research: The DIWHY study is an anonymous online survey. The first part of this online survey consists of 34 multiple choice questions. Additionally, you are invited to reflect on your motivations behind DIY-looping, to share your individual patient journey, and to describe any changes you have experienced in your day-to-day lives. Furthermore, we ask for your self-reported, basic medical information and some relevant personal data (age, sex, country of origin, duration of diabetes, start date using a DIY APS). The survey runs on the secure platform RedCap, which has been established for scientific surveys.

Possible benefit for the study participant or for the general public: As part of the EU-H2020 funded "OPEN"-project (Outcomes of Patients' Evidence with Novel, Do-It-Yourself Artificial Pancreas Technology), this study will provide a better understanding of the unmet needs of people with diabetes and current challenges to uptake, which will, in turn, facilitate dialogue and collaboration to strengthen the involvement of open source approaches in healthcare. In the future, people with diabetes can benefit significantly from the earliest possible establishment of approved closed-loop systems. To learn more about the OPEN project, please visit: http://www.open-diabetes.eu

Possible risks: There are no foreseeable risks and disadvantages of participation. The survey is anonymous.

Reimbursement: Your participation in the study is free.

Circumstances that may lead to the termination of study participation: Subsequent deletion of your personal data record is not possible for anonymity reasons. Therefore, it is not possible to drop out after the survey has been submitted. After the analysis has been completed, all data will be deleted.

Voluntary Partipication: Your participation is voluntary.

Insurance: No special insurance for the patients was completed for this study.

Data protection: All data collected in this study underlie the federal and local laws of data protection. Through your declaration of consent, you agree that the study doctors and scientific staff may use your personal data exclusively for the purpose of this study. Personal data is e.g. age, gender, basic medical data or other personal information collected during your participation in the survey. Your data will be processed as an anonymous record for research purposes for statistical evaluation. Please note that the results of the study can be published in the medical literature, however, no conclusions to your person are possible.

Right to ask questions: You have the right to ask your questions to the study team at any time about all matters concerning the study, in particular about risks.

Who to contact

Pricipal Investigator:
Dr. med. Katarina Braune
Charité - Universitätsmedizin Berlin
Campus Virchow Klinikum (CVK)
Klinik für Pädiatrie m.S. Endokrinologie und Diabetologie
Augustenburger Platz 1
13353 Berlin
Germany
Phone: +4930/450616454
Email: katarina.braune@charite.de

Ethics/IRB

This proposal has been reviewed and approved by the ethics committee of Charité – Universtätsmedizin Berlin, Germany, which is a committee whose task it is to make sure that research participants are protected from harm. If you wish to find about more about the IRB, contact the chair of the ethics committee office, Dr. Katja Orzechowski, Campus Charité Mitte, Charitéplatz 1,10117 Berlin, Germany, phone: +49 30 450 517 222).

Thank you very much for your participation!
The OPEN study team

☐ I hereby give my consent to participate in this study.

Please specify your child's gender.	◯ Female<br>◯ Male<br>◯ Other
Please enter your child's age in years.	__________________________

Please enter your child's country of residence.

- ◯ I'd rather not say
- ◯ Afghanistan
- ◯ Albania
- ◯ Algeria
- ◯ American Samoa
- ◯ Andorra
- ◯ Angola
- ◯ Anguilla
- ◯ Antarctica
- ◯ Antigua and Barbuda
- ◯ Argentina
- ◯ Armenia
- ◯ Aruba
- ◯ Australia
- ◯ Austria
- ◯ Azerbaijan
- ◯ Bahamas
- ◯ Bahrain
- ◯ Bangladesh
- ◯ Barbados
- ◯ Belarus
- ◯ Belgium
- ◯ Belize
- ◯ Benin
- ◯ Bermuda
- ◯ Bhutan
- ◯ Bolivia
- ◯ Bosnia and Herzegovina
- ◯ Botswana
- ◯ Bouvet Island
- ◯ Brazil
- ◯ British Indian Ocean Territory
- ◯ Brunei
- ◯ Bulgaria
- ◯ Burkina Faso
- ◯ Burundi
- ◯ Cambodia
- ◯ Cameroon
- ◯ Canada
- ◯ Cape Verde
- ◯ Cayman Islands
- ◯ Central African Republic
- ◯ Chad
- ◯ Chile
- ◯ China
- ◯ Christmas Island
- ◯ Cocos (Keeling) Islands
- ◯ Colombia
- ◯ Comoros
- ◯ Congo
- ◯ Cook Islands
- ◯ Costa Rica
- ◯ Côte d'Ivoire
- ◯ Ivory Coast
- ◯ Croatia
- ◯ Cuba
- ◯ Cyprus
- ◯ Czech Republic
- ◯ Denmark
- ◯ Djibouti
- ◯ Dominica
- ◯ Dominican Republic
- ◯ Ecuador
- ◯ Egypt
- ◯ El Salvador
- ◯ Equatorial Guinea
- ◯ Eritrea
- ◯ Estonia
- ◯ Ethiopia

◯ Falkland Islands (Malvinas)
◯ Faroe Islands
◯ Fiji
◯ Finland
◯ France
◯ French Guiana
◯ French Polynesia
◯ French Southern Territories
◯ Gabon
◯ Gambia
◯ Georgia
◯ Germany
◯ Ghana
◯ Gibraltar
◯ Greece
◯ Greenland
◯ Grenada
◯ Guadeloupe
◯ Guam
◯ Guatemala
◯ Guernsey
◯ Guinea
◯ Guinea-Bissau
◯ Guyana
◯ Haiti
◯ Heard Island and McDonald Islands
◯ Holy See (Vatican City State)
◯ Honduras
◯ Hong Kong
◯ Hungary
◯ Iceland
◯ India
◯ Indonesia
◯ Islamic Republic of
◯ Iraq
◯ Ireland
◯ Isle of Man
◯ Israel
◯ Italy
◯ Jamaica
◯ Japan
◯ Jersey
◯ Jordan
◯ Kazakhstan
◯ Kenya
◯ Kiribati
◯ Korea (North)
◯ Korea (South)
◯ Kuwait
◯ Kyrgyzstan
◯ Lao People's Democratic Republic
◯ Latvia
◯ Lebanon
◯ Lesotho
◯ Liberia
◯ Libya
◯ Liechtenstein
◯ Lithuania
◯ Luxembourg
◯ Macao
◯ Macedonia
◯ Madagascar
◯ Malawi
◯ Malaysia
◯ Maldives
◯ Mali
◯ Malta
◯ Marshall Islands
◯ Martinique
◯ Mauritania
◯ Mauritius

◯ Mayotte
◯ Mexico
◯ Micronesia
◯ Moldova
◯ Monaco
◯ Mongolia
◯ Montenegro
◯ Montserrat
◯ Morocco
◯ Mozambique
◯ Myanmar
◯ Burma
◯ Namibia
◯ Nauru
◯ Nepal
◯ Netherlands
◯ Netherlands Antilles
◯ New Caledonia
◯ New Zealand
◯ Nicaragua
◯ Niger
◯ Nigeria
◯ Niue
◯ Norfolk Island
◯ Northern Mariana Islands
◯ Norway
◯ Oman
◯ Pakistan
◯ Palau
◯ Palestine
◯ Panama
◯ Papua New Guinea
◯ Paraguay
◯ Peru
◯ Philippines
◯ Pitcairn
◯ Poland
◯ Portugal
◯ Puerto Rico
◯ Qatar
◯ Réunion
◯ Romania
◯ Russia
◯ Rwanda
◯ Saint Helena, Ascension and Tristan da Cunha
◯ Saint Kitts and Nevis
◯ Saint Lucia
◯ Saint Pierre and Miquelon
◯ Saint Vincent and the Grenadines
◯ Samoa
◯ San Marino
◯ Sao Tome and Principe
◯ Saudi Arabia
◯ Senegal
◯ Serbia
◯ Seychelles
◯ Sierra Leone
◯ Singapore
◯ Slovakia
◯ Slovenia
◯ Solomon Islands
◯ Somalia
◯ South Africa
◯ South Georgia and the South Sandwich Islands
◯ Spain
◯ Sri Lanka
◯ Sudan
◯ Suriname
◯ Svalbard and Jan Mayen
◯ Swaziland
◯ Sweden

◯ Switzerland
◯ Syria
◯ Taiwan
◯ Tajikistan
◯ Tanzania
◯ Thailand
◯ Timor-Leste
◯ Togo
◯ Tokelau
◯ Tonga
◯ Trinidad and Tobago
◯ Tunisia
◯ Turkey
◯ Turkmenistan
◯ Turks and Caicos Islands
◯ Tuvalu
◯ Uganda
◯ Ukraine
◯ United Arab Emirates
◯ United Kingdom
◯ United States
◯ United States Minor Outlying Islands
◯ Uruguay
◯ Uzbekistan
◯ Vanuatu
◯ Venezuela
◯ Vietnam
◯ Virgin Islands, British
◯ Virgin Islands, U.S.
◯ Wallis and Futuna
◯ Western Sahara
◯ Yemen
◯ Zambia
◯ Zimbabwe

Please enter your child's country of origin.

◯ I'd rather not say
◯ Afghanistan
◯ Albania
◯ Algeria
◯ American Samoa
◯ Andorra
◯ Angola
◯ Anguilla
◯ Antarctica
◯ Antigua and Barbuda
◯ Argentina
◯ Armenia
◯ Aruba
◯ Australia
◯ Austria
◯ Azerbaijan
◯ Bahamas
◯ Bahrain
◯ Bangladesh
◯ Barbados
◯ Belarus
◯ Belgium
◯ Belize
◯ Benin
◯ Bermuda
◯ Bhutan
◯ Bolivia
◯ Bosnia and Herzegovina
◯ Botswana
◯ Bouvet Island
◯ Brazil
◯ British Indian Ocean Territory
◯ Brunei
◯ Bulgaria
◯ Burkina Faso
◯ Burundi
◯ Cambodia
◯ Cameroon
◯ Canada
◯ Cape Verde
◯ Cayman Islands
◯ Central African Republic
◯ Chad
◯ Chile
◯ China
◯ Christmas Island
◯ Cocos (Keeling) Islands
◯ Colombia
◯ Comoros
◯ Congo
◯ Cook Islands
◯ Costa Rica
◯ Côte d'Ivoire
◯ Ivory Coast
◯ Croatia
◯ Cuba
◯ Cyprus
◯ Czech Republic
◯ Denmark
◯ Djibouti
◯ Dominica
◯ Dominican Republic
◯ Ecuador
◯ Egypt
◯ El Salvador
◯ Equatorial Guinea
◯ Eritrea
◯ Estonia
◯ Ethiopia

◯ Falkland Islands (Malvinas)
◯ Faroe Islands
◯ Fiji
◯ Finland
◯ France
◯ French Guiana
◯ French Polynesia
◯ French Southern Territories
◯ Gabon
◯ Gambia
◯ Georgia
◯ Germany
◯ Ghana
◯ Gibraltar
◯ Greece
◯ Greenland
◯ Grenada
◯ Guadeloupe
◯ Guam
◯ Guatemala
◯ Guernsey
◯ Guinea
◯ Guinea-Bissau
◯ Guyana
◯ Haiti
◯ Heard Island and McDonald Islands
◯ Holy See (Vatican City State)
◯ Honduras
◯ Hong Kong
◯ Hungary
◯ Iceland
◯ India
◯ Indonesia
◯ Islamic Republic of
◯ Iraq
◯ Ireland
◯ Isle of Man
◯ Israel
◯ Italy
◯ Jamaica
◯ Japan
◯ Jersey
◯ Jordan
◯ Kazakhstan
◯ Kenya
◯ Kiribati
◯ Korea (North)
◯ Korea (South)
◯ Kuwait
◯ Kyrgyzstan
◯ Lao People's Democratic Republic
◯ Latvia
◯ Lebanon
◯ Lesotho
◯ Liberia
◯ Libya
◯ Liechtenstein
◯ Lithuania
◯ Luxembourg
◯ Macao
◯ Macedonia
◯ Madagascar
◯ Malawi
◯ Malaysia
◯ Maldives
◯ Mali
◯ Malta
◯ Marshall Islands
◯ Martinique
◯ Mauritania
◯ Mauritius

◯ Mayotte
◯ Mexico
◯ Micronesia
◯ Moldova
◯ Monaco
◯ Mongolia
◯ Montenegro
◯ Montserrat
◯ Morocco
◯ Mozambique
◯ Myanmar
◯ Burma
◯ Namibia
◯ Nauru
◯ Nepal
◯ Netherlands
◯ Netherlands Antilles
◯ New Caledonia
◯ New Zealand
◯ Nicaragua
◯ Niger
◯ Nigeria
◯ Niue
◯ Norfolk Island
◯ Northern Mariana Islands
◯ Norway
◯ Oman
◯ Pakistan
◯ Palau
◯ Palestine
◯ Panama
◯ Papua New Guinea
◯ Paraguay
◯ Peru
◯ Philippines
◯ Pitcairn
◯ Poland
◯ Portugal
◯ Puerto Rico
◯ Qatar
◯ Réunion
◯ Romania
◯ Russia
◯ Rwanda
◯ Saint Helena, Ascension and Tristan da Cunha
◯ Saint Kitts and Nevis
◯ Saint Lucia
◯ Saint Pierre and Miquelon
◯ Saint Vincent and the Grenadines
◯ Samoa
◯ San Marino
◯ Sao Tome and Principe
◯ Saudi Arabia
◯ Senegal
◯ Serbia
◯ Seychelles
◯ Sierra Leone
◯ Singapore
◯ Slovakia
◯ Slovenia
◯ Solomon Islands
◯ Somalia
◯ South Africa
◯ South Georgia and the South Sandwich Islands
◯ Spain
◯ Sri Lanka
◯ Sudan
◯ Suriname
◯ Svalbard and Jan Mayen
◯ Swaziland
◯ Sweden

	◯ Switzerland<br>◯ Syria<br>◯ Taiwan<br>◯ Tajikistan<br>◯ Tanzania<br>◯ Thailand<br>◯ Timor-Leste<br>◯ Togo<br>◯ Tokelau<br>◯ Tonga<br>◯ Trinidad and Tobago<br>◯ Tunisia<br>◯ Turkey<br>◯ Turkmenistan<br>◯ Turks and Caicos Islands<br>◯ Tuvalu<br>◯ Uganda<br>◯ Ukraine<br>◯ United Arab Emirates<br>◯ United Kingdom<br>◯ United States<br>◯ United States Minor Outlying Islands<br>◯ Uruguay<br>◯ Uzbekistan<br>◯ Vanuatu<br>◯ Venezuela<br>◯ Vietnam<br>◯ Virgin Islands, British<br>◯ Virgin Islands, U.S.<br>◯ Wallis and Futuna<br>◯ Western Sahara<br>◯ Yemen<br>◯ Zambia<br>◯ Zimbabwe
Please enter your own occupational status.	◯ Full time<br>◯ Part time<br>◯ Unemployed<br>◯ Retired<br>◯ Student<br>◯ None of the above / I'd rather not say
Please enter your own job title.	______________________________<br>(If you'd rather not say, just leave this field open.)
Please enter your household's annual net income. Please convert your local currency to US-Dollar for comparision.	◯ under 20 000 USD<br>◯ 20 000 to 34 999 USD<br>◯ 35 000 to 49 999 USD<br>◯ 50 000 to 74 999 USD<br>◯ 75 000 to 99 999 USD<br>◯ over 100 000 USD<br>◯ I'd rather not say

What is the highest degree or level of school you have completed? If you are currently enrolled, please provide the highest degree received.	◯ No schooling completed<br>◯ Nursery school to 8th grade<br>◯ Some high school (no diploma)<br>◯ High school graduate (diploma or the equivalent)<br>◯ Some college credit (no degree)<br>◯ Trade/technical/vocational training<br>◯ Associate degree<br>◯ Bachelor's degree<br>◯ Master's degree<br>◯ Professional degree<br>◯ Doctorate degree<br>◯ None of the above / I'd rather not say
Please specify what type of diabetes applies to your child.	◯ Type 1 diabetes<br>◯ Type 2 diabetes<br>◯ Gestational diabetes<br>◯ Other<br>◯ I don't know
Please enter your child's date of diagnosis. (D-M-Y)	__________________________________<br>(If you are unsure, please give an estimate or leave this field free.)

Please enter the date when your child started using DIY APS. (D-M-Y)	________________________________ (If you are unsure, please give an estimate or leave this field free.)
Which type of DIY APS does your child use regularly? Multiple options are possible.	☐ OpenAPS<br>☐ AndroidAPS<br>☐ Loop<br>☐ Other<br>☐ I don't know / I'd rather not say
If you are using an "other" system, please specify what system your child is using.	________________________________
Please tell us the approximate amount you have to pay each year for extra technology (such as server costs, hardware) to make DIY APS possible. Please convert your local currency to US-Dollar for comparision.	________________________________

If available, please give your child's last 3 HbA1c results from BEFORE DIY APS was used regularly.

Last HbA1c result from before DIY APS was started:

____
(The dimension unit is automatically set to "%". Please use a dot as decimal separator and only enter the number, e.g. "7.5". If you are unsure, please leave this field free.)

Second-last HbA1c result from before DIY APS was started:

____
(The dimension unit is automatically set to "%". Please use a dot as decimal separator and only enter the number, e.g. "7.5". If you are unsure, please leave this field free.)

Third-last HbA1c results from before DIY APS was started:

____
(The dimension unit is automatically set to "%". Please use a dot as decimal separator and only enter the number, e.g. "7.5". If you are unsure, please leave this field free.)

If available, please give your child's Time in Range (percentage of sensor glucose within 70 - 180 mg/dl or 3,9 - 10,0 mmol/l) from BEFORE DIY APS was used regularly.

____
(The dimension unit is automatically set to "%". Please use a dot as decimal separator and only enter the number, e.g. "70.5". If you are unsure, please leave this field free.)

If available, please give your child's first 3 HbA1c results from AFTER DIY APS was used regularly.

First HbA1c result AFTER the start of DIY APS:

____
(The dimension unit is automatically set to "%". Please use a dot as decimal separator and only enter the number, e.g. "7.5". If you are unsure, please leave this field free.)

Second HbA1c result AFTER the start of DIY APS:

____
(The dimension unit is automatically set to "%". Please use a dot as decimal separator and only enter the number, e.g. "7.5". If you are unsure, please leave this field free.)

Third HbA1c result AFTER the start of DIY APS.

____
(The dimension unit is automatically set to "%". Please use a dot as decimal separator and only enter the number, e.g. "7.5". If you are unsure, please leave this field free.)

If available, please give your child's Time in Range (percentage of sensor glucose within 70 - 180 mg/dl or 3,9 - 10,0 mmol/l) from AFTER DIY APS was used regularly.

____
(The dimension unit is automatically set to "%". Please use a dot as decimal separator and only enter the number, e.g. "70.5". If you are unsure, please leave this field free.)

What motivated you to build a Do-it-yourself Artificial Pancreas system for your child? Indicate your level of agreement with each statement by ticking one of the responses.

I built a DIY APS ...

	Fully applies	Largely applies	Partially applies	Does rather not apply	Does not apply at all
To achieve better overall glycaemic control.	○	○	○	○	○
To reduce the occurrence of acute complications (such as hypoglycaemia and hyperglycaemia).	○	○	○	○	○
To reduce the long-term complication risk (such as diabetic nephropathy, neuropathy, retinopathy etc.).	○	○	○	○	○
To put diabetes management more on auto-pilot and interact less frequently with the therapy system.	○	○	○	○	○
To improve my child's sleep quality.	○	○	○	○	○
To improve sleep quality of partner/parents/family.	○	○	○	○	○
To increase my child's life expectancy.	○	○	○	○	○
Due to the lack of availability of commercial closed loop systems in my country.	○	○	○	○	○
Due to the costs of commercially available closed loop systems (too expensive or not reimbursed by the healthcare system).	○	○	○	○	○
As commercially available closed loop systems are not suitable for my child's individual needs.	○	○	○	○	○
As therapy goals could not be achieved with the available therapy options.	○	○	○	○	○
Due to lack of medical support from the diabetes team (endocrinologist, diabetes educator, nurse, dietitian).	○	○	○	○	○

Due to lack of psychosocial support from health care professionals.	◯	◯	◯	◯	◯
Out of curiosity (technical or medical interest).	◯	◯	◯	◯	◯

Main other reason(s) (if any)

____
(max 500 words)

If you would like, please share your personal story about why you decided to build your own artificial pancreas system and how you got started. Feel free to share any experiences that had a significant impact on how you manage your diabetes as well. This story can be as short or as long as you wish.

When reflecting on your personal DIY closed loop story, you may want to consider the following:
- When did you first hear about DIY closed loop systems and how did you look for further information?
- Were there any key events or experiences that were a factor in your decision to begin closed looping?
- Was there anyone else involved in helping you come to decision to begin DIY closed looping? For example a friend, family member or an online support group?
- What were your emotions in the lead up to building your DIY closed loop system? For example, had you any major hopes or fears?

____
(max 1000 words)

Finally, describe some of the changes (if any) you experienced in your day-to-day life following successfully building the closed loop system. Did you experience any difficulties in making the transition to DIY closed looping?

____
(max 1000 words)

You can submit your answers by clicking the "submit" button below. Thank you very much for your participation!
The OPEN research consortium.
