# Supplementary material for: Why #WeAreNotWaiting—Motivations and Self-Reported Outcomes Among Users of Open-source Automated Insulin Delivery Systems: Multinational Survey
Source: J Med Internet Res. 2021 Jun 7;23(6):e25409. doi: 10.2196/25409 (PMC8218212; doi:10.2196/25409)
Supplement: Multimedia Appendix 3 [file jmir_v23i6e25409_app3.docx]

**Suppl. Table 1:** Codes and frequency of mentioned additional motivations as responses to the open-ended question.

| **Code** | **Caregivers of children and adolescents with diabetes** [%] | **Adults**  **with diabetes** [%] | **All** [%] |
| --- | --- | --- | --- |
| Autonomy* | 41.7 | 22.3 | 26.0 |
| Improving diabetes management | 45.8 | 46.6 | 46.5 |
| Community spirit* | 4.2 | 13.6 | 11.8 |
| Comorbidities* | 4.2 | 10.7 | 9.5 |
| Curiosity | 0.0 | 10.7 | 8.7 |
| Diabetes distress/burnout* | 4.2 | 6.8 | 6.3 |
| Diabetes-related complications | 0.0 | 5.8 | 4.7 |
| Dissatisfaction with available technology, choice and healthcare | 20.8 | 24.3 | 23.6 |
| Features only available as DIY | 37.5 | 14.6 | 18.9 |
| Remote management*†* | 20.8 | 0.0 | 3.9 |
| Female health* | 0.0 | 6.8 | 5.5 |
| Improving performance* | 0.0 | 5.8 | 4.7 |
| DIY mindset/early adopter of technology* | 4.2 | 21.4 | 18.1 |
| Normal life/less burden | 45.8 | 34.0 | 36.2 |
| Out-of-pocket expenses | 8.3 | 2.9 | 3.9 |
| Other | 8.3 | 8.7 | 8.7 |
| Quality of sleep | 16.7 | 13.6 | 14.2 |
| Safety/reducing severe hypoglycemia | 0.0 | 10.7 | 8.7 |

** Motivations additional to the survey questions*

*† Motivations highlighted by parents/caregivers only*

**Suppl. Table 2:** Detail of Time-in-Range (TIR) improvements following open-source AID implementation.

| **Population** | **N** | **Mean TIR before open-source AID**  [% ±SD] | **Mean TIR with open-source AID**  [% ±SD] | **Wilcoxon Test** | | **Individuals with Increased TIR** [%] |
| --- | --- | --- | --- | --- | --- | --- |
|  |  |  |  | **P-value** | **Effect size** [%] |  |
| All | 365 | 62.96 ±16.18 | 80.34 ±9.41 | < 2.2e-16 | +17.38 | 95.62 |
| Adults | 310 | 63.00 ±16.40 | 80.30 ±9.45 | 1.074e-10 | +17.30 | 96.16 |
| Children and Adolescents | 55 | 62.60 ±15.30 | 80.70 ±9.26 | < 2.2e-16 | +18.10 | 98.18 |
